# Academy of Stomatology

**Published:** 1903-02

**Authors:** 

**Affiliations:** Academy of Stomatology


### Reports of Society Meetings.

### ACADEMY OF STOMATOLOGY.

A REGULAR meeting of the Academy of Stomatology, of Philadelphia, was held at its rooms, 1731 Chestnut Street, on the evening of Tuesday, June 24, 1902, the President, Dr. R. Hamil D. Swing, in the chair.

A paper entitled "The Regeneration of the Pulp" was read by Dr. Arthur R. Dray.

(For Dr. Dray's paper, see page 81.)

### DISCUSSION.

*Dr. E. T. Darby.*—The paper is certainly a novel and interesting one. There is much in it that is new to me. I did not suppose that pulps that had been extirpated could regenerate and fill the pulp-chamber. It was the practice of old Dr. Maynard to simply cover over the canal openings after pulp-extirpation, and he claimed that he had as much success from that method as he had in the later years when he filled the pulp-canals. One would now question very much the wisdom of putting a diaphragm between the pulp-chamber and the cavity of decay, leaving the pulp-canal and chamber unoccupied. I have never yet seen an instance in which I thought the pulp renewed itself. I have never opened into the canal of a tooth and found it filled with pulp after the extirpation of the original pulp. If I understand the essayist aright, he finds vital pulps, pulps that may be exposed the second time, that may bleed, and that are highly sensitive, just as when the pulp was originally exposed by accident. If such conditions exist, I have certainly learned something to-night.

*Dr. E. C. Kirk.*—As Dr. Darby has said, this is an absolutely novel idea to me. If a man should come here and say that after an amputation of an arm he had seen the whole arm regenerated, I should as readily believe the statement as that which I have heard this evening. The pulp of a tooth is not simply a tissue. It is a very complex organization, and I doubt, to put it mildly, whether such an organ is ever reproduced in the human being after it is once extirpated. I want to say this reverently, because here is a *confrère* who, with evident honesty of purpose, reports the results of his observations, and I honor him for his courage in doing it. Even though I say I do not believe that what he says is true, I do not in the slightest degree intimate that he is making a misstatement, but, judging from my own experience, I think he has made an error of observation. This amounts to a difference of opinion.

I would like to go over the cases he reports and see upon what phenomena he bases such an astounding statement. I can to some extent follow him in his case where he has extirpated the pulp and yet found some sort of tissue in the pulp-chamber after an aseptic operation, but when he reports a similar result in a case of infection of the apical tissue, that is beyond my ability to believe. I think he has made a mistake, yet I do not wish to discredit his position, because he has an evident honesty of purpose, and he certainly has seen something that leads him to this conclusion. We do not yet know all we should know about the dental pulp.

I once had a woman patient, about fifty years of age, who had lost a central incisor by resorption of the root, and when the tooth was removed I split it open and found the whole pulp-chamber completely filled with a calcified mass. In the woman's younger days the tooth had been knocked out and had been replanted. The tooth was dislocated at a period of life when the pulp-chamber should be large, and yet before the loss of the tooth this deposition of secondary dentine must have taken place. I have always been at a loss to account for the condition. We cannot have the calcific deposit unless there is some vital union between the pulp in the chamber and the apical tissues which supply it nutrition. Dr. Dray's observations are extremely interesting, and are of such a character that I think they should be further investigated. I understand that he based his diagnosis of pulp regeneration in his infectious case upon the fact of thermal reaction. This is likely to be misleading unless very carefully done. The removal of the pulp a second time from a tooth has no counterpart in my experience, nor in anything I have ever read on the subject.

*Dr. H. B. Hickman.*—If the pulp were in truth a nerve, I feel that it might possibly regenerate. I do not believe that the temperature test can be relied upon. At the present time I have a patient who had suffered greatly from the sensitiveness of his teeth to cold. I devitalized the affected ones, and now when he eats sweet things, or cold substances strike them, he has pain. The man seems to have an ordinary amount of intelligence, and I do not think would deceive me. The pulps have certainly not regenerated, because the canals are filled with oxychloride of zinc. Nevertheless, there was decided sensitivity after their removal and until zinc chloride was applied about the necks of the teeth from which the gum had receded.

*Dr. S. H. Guilford.*—I am glad that the essayist has come forward and presented what he believes to be new, and while I admire the courage of his convictions, I do not think his convictions are right. It seems to me that in this case he has pretty largely jumped at conclusions, and that he has not had sufficient data upon which to base them. If I understand him correctly, he had five cases, three of which were successful and two were failures, which, I take it, was too small a number upon which to base anything in this line of work. If we analyze his work we will find that in two of these cases which he counts successful there was no pain. He does not tell us that he afterwards opened into the pulp-canal to ascertain whether the pulp was alive, but he infers that there was regeneration of tissue because the patient experienced no trouble. In the third case he infers that there was regeneration because, upon going into the chamber a second time there was a free flow of blood and considerable pain. I think we can account for this in some other way. In just what condition the pulp-chamber may have been in in this third case we do not know, but we do know that frequently the vessels of the pulp become congested, and we may have a fungous growth of the pulp which is engorged with blood, and which when touched causes profuse hemorrhage. The only way in which to decide whether Dr. Dray's views are correct would be to extract the tooth, open and examine it. The fact that the tooth was sensitive to the instrument is not proof, because I have seen cases in which teeth that have been devitalized have appeared to be sensitive to the instrument. None of us have ever supposed that under such conditions the pulp-tissue had been reformed. I do not see how it can occur, because we have many elements entering into the composition of the pulp. In order to have a pulp we would have to have something more than a portion of the nerve itself. I think that by a little closer investigation the essayist will find that he was greatly deceived. We believe that the best treatment is to sterilize the canal and fill it as completely as possible in order to prevent future trouble. That a pulp-canal may be left vacant, covered over, and not give trouble is entirely possible. I would not consider it good practice, but that it might happen I can readily conceive. In regard to formation of new pulp-tissue, however, I do not think that such a thing is at all possible.

*Dr. Kirk.*—I do not think we know all we should upon the subject of sensitivity. It is quite possible that in these cases where we have extirpated the pulp we may have sensitivity of the dentine because of the passage of fibres from the pericementum into the dentine. To what extent this may occur we do not know, as it varies in different specimens.

The most curious thing to me is the essayist's report of finding something like pulp-tissue that was sensitive and vascular in a tooth from which he had extracted the original pulp. He does not say that it was a pulp organized as we understand it, but he found something that indicated regeneration in two ways,—in vascularity and in sensitiveness. I think the subject is worthy of further investigation, and should be made the subject of careful scientific study. The only way to decide the point is, as Dr. Guilford has said, to make a post-mortem examination of one of these teeth. I cannot agree with Dr. Guilford that the observation is valueless because it is not based upon a larger number of cases. One such case proves the point if it is at all successfully demonstrated.

*Dr. J. E. Libbey.*—Though I am somewhat sceptical, I have come to feel that I am not ready to taboo anything until it is disproved. I thought, when the essayist was speaking, of how years ago it was said of the man who invented illuminating gas, that a poor Dutchman was trying to light the city of London with smoke. Well, the Dutchman succeeded. This idea of regeneration of a pulp after its extraction is so entirely new to me that I cannot combat it. I never saw a case of the kind, and it is the first case I ever heard of. I hope these investigations will go farther and that we may learn something. The matter of capping over and leaving empty the pulp-chamber and canal and its remaining perfectly comfortable for even a long time I can understand. We know that after filling, in many cases the pulps die, sometimes without any irritation whatever. I think few dentists like to open into one of those canals after it has been lying dormant for a long time. I have opened into them time and again, and could detect no odor, but I would expect under the old method of treatment to have trouble inside of the next twenty-four hours. I think further investigation will prove that there was not regeneration of the pulp. In regeneration there must have been previous breaking down of tissue. All histology teaches us that there are no lymphatics of the pulp. If there is a breaking down, and no means of carrying away the broken-down tissue, how can we expect regeneration? To me it at present seems an impossibility.

*Dr. A. N. Gaylord.*—I have the same feeling of great surprise expressed by the other gentlemen. The subject is something entirely new to me, and yet I think that if a few years ago some one had told us we could talk to a person several miles distant and be heard distinctly, we would have accepted the statement with equal hesitancy. I think this case as stated is improbable. There may be a possibility that this lateral incisor had two roots, and that the pulp was extracted at first from but one root, and that the second extraction was from the other root.

*Dr. J. H. Gaskill.*—I have been rather interested, but I cannot help saying that I doubt the correctness of the observations of the essayist. Possibly the appearance of regeneration might be explained in several other ways. I think we have had sufficient evidence offered to know that the pulp can resorb the tooth substance. I have had a number of such cases come into my practice. In one case the absorption had so affected the dentine below the gum margin that there was difficulty in removing the pulp. As to the return of sensitiveness to the molar tooth from which the pulp was extracted, I may say that the anæsthesia is sometimes so complete that there may be failure to extract the entire pulp, and owing to some portions remaining at the apex the tooth may later respond to thermal tests. I think it would be well worth while to explore a little farther into the condition of this tooth and see whether there has been any opening below the cavity.

*Dr. W. H. Trueman.*—The paper of the essayist reminds me of two instances in my own personal experience. The pulp of one of my central incisors was devitalized and extracted and the canal filled with cotton and creosote and allowed to rest for two weeks. During that interval I did not know I had a tooth. I reported to the dentist at the proper time, and he removed the cotton. At the moment he placed the instrument inside the cavity it felt as though there were ten thousand vital nerves there. The outside of the tooth was not sensitive. There was nothing to be done but to close up the tooth and allow it to rest for some time, when the root-filling was completed without the slightest inconvenience. It remained quiet for some time, but later began to give me trouble. Several examinations were made by different men. Finally Dr. Marshall Webb drilled into the tooth and found a mummified pulp. After removing this he treated the tooth, and for fifteen years it has remained perfectly comfortable.

While it is not safe to say that a pulp cannot regenerate, all knowledge we have seems to point to the conclusion that the idea is born of a mistake. We cannot tell in every case whether a tooth is vital or not. We may find a tooth which we know to be vital, yet does not respond to heat and cold; again, the reverse is observed. A case has been reported of an extracted tooth which was replaced and became quite firm. After some years, while a cavity in it was being excavated for filling, it was found to be sensitive. The conclusion was that there had been a reunion of the vessels of the pulp and apical tissues. It seems to me it would be wise to hold the matter under consideration, and to remember what Dr. Miller said, that it takes at least ten years to express an opinion upon any new method of pulp treatment. It is well to go slowly.

*Dr. Dray.*—I am exceedingly glad that I did make the statement, for it has brought out many points of interest. I do not feel that it is for me to challenge what I have been told by more experienced men, but I do challenge the remark made by one of the gentlemen this evening, that nervous tissue is the only tissue that can regenerate. We can also have regeneration of blood-vessels, of connective tissue, and the organization of a blood-clot. This is a possible occurrence in a devitalized tooth, whether it is really pulp-tissue that is formed or not. I gave the history of the structure found in the tooth from which I had extracted the pulp. Something certainly had regenerated. It was more sensitive, more vascular, and the covering was different from that on the original pulp. I cited only five cases, but, as was stated this evening, one case if proved is a sufficient index to other possible cases. The lateral incisor did not have sensitive dentine, but did have this secondary growth, whatever it may have been. It bore a strong resemblance to the original pulp, but that does not count. It was, however, something tangible. A fungous pulp I also think indicates a regenerative possibility. I thank you for your kindly criticism, which has added data that will aid me in future observations.

Adjourned.

Otto E. Inglis, D.D.S.,
*Editor Academy of Stomatology.*

